# When promotion becomes a bounty on publications: how academic reward systems shape publication behavior, with lessons from Nepal

**DOI:** 10.1097/MS9.0000000000005602

**Published:** 2026-09-08

**Authors:** Naveen Gautam

**Affiliations:** Institute of Medicine, Tribhuvan University, Kathmandu, Nepal

**Keywords:** academic reward systems, bibliometrics, DORA, low- and middle-income countries, publication ethics, research assessment

## Abstract

The criteria institutions use to appoint and promote academics shape how science is produced. Where advancement is tied tightly to publication counts, researchers respond rationally to the incentive, and publication behavior shifts toward volume. This article uses the faculty-promotion regulations of Tribhuvan University, Nepal’s largest higher education institution, as a worked example of how publication-weighted criteria may reward output over scientific merit. The framework allocates the majority of open-competition marks to research publications, sets minimum article counts for promotion, fractionates credit by authorship position, and scores articles by journal tier. Such a structure illustrates a problem common across many low- and middle-income academic systems: it can encourage fragmented, lower-quality output, penalize collaborative authorship, and use journal-based metrics as surrogates for individual article quality, contrary to the San Francisco Declaration on Research Assessment. The institution’s existing anti-plagiarism and anti-predatory safeguards show that meaningful reform is achievable. Drawing on DORA, the International Committee of Medical Journal Editors authorship criteria, and Committee on Publication Ethics guidance, the article argues for a phased shift toward portfolio-based assessment that rewards the quality, integrity, and relevance of scholarship rather than the counting of papers.

## Background

Across the global research enterprise, the criteria that institutions use to appoint and promote academics quietly determine how science is produced. Where advancement is tied tightly to publication counts, researchers respond rationally to the incentive in front of them, and publication behavior is reshaped accordingly. A growing literature documents how reward systems built on bibliometric thresholds can encourage volume over substance, particularly in resource-constrained settings where funding and mentorship are scarce[1]. In South Asia, the coupling of rigid bibliometric thresholds to faculty promotion has been linked to questionable research practices, including salami slicing, honorary authorship, and submissions to predatory journals^[^2,3^]^.

This article examines how one such reward system operates in practice and what it implies for research integrity more broadly. Tribhuvan University (TU), Nepal’s largest higher education institution, provides an instructive worked example of how publication-weighted promotion criteria may inadvertently reward output volume over scientific merit. The mechanism it illustrates, rather than the institution itself, is the focus of interest: TU’s framework is one concrete instance of a structure replicated across many low- and middle-income academic systems.

## The mechanism: a worked example

The TU Teacher Appointment Recommendation Regulations, 2075 (amended through 2081 BS), stipulate that open-competition candidates for Professor must hold a PhD or DM/MCh, possess 10 years of teaching experience, and have published at least 10 research articles in recognized journals, with at least five as Principal Author[4]. Associate Professor candidates require six articles, three as Principal Author. Critically, the open-competition scoring rubric allocates 70 of 100 marks directly to research publications, with only 20 marks for teaching experience and 10 for interview performance (Fig. 1)[4]. Publications are scored through a journal-tier system (Schedule 5: Classes A–D) based on indexing databases such as SCImago and PubMed, and marks are fractionated by authorship order: Principal Authors receive full marks, Corresponding Authors receive 70%, and the remaining co-authors share the residual points pro rata[4].

**Figure 1.**
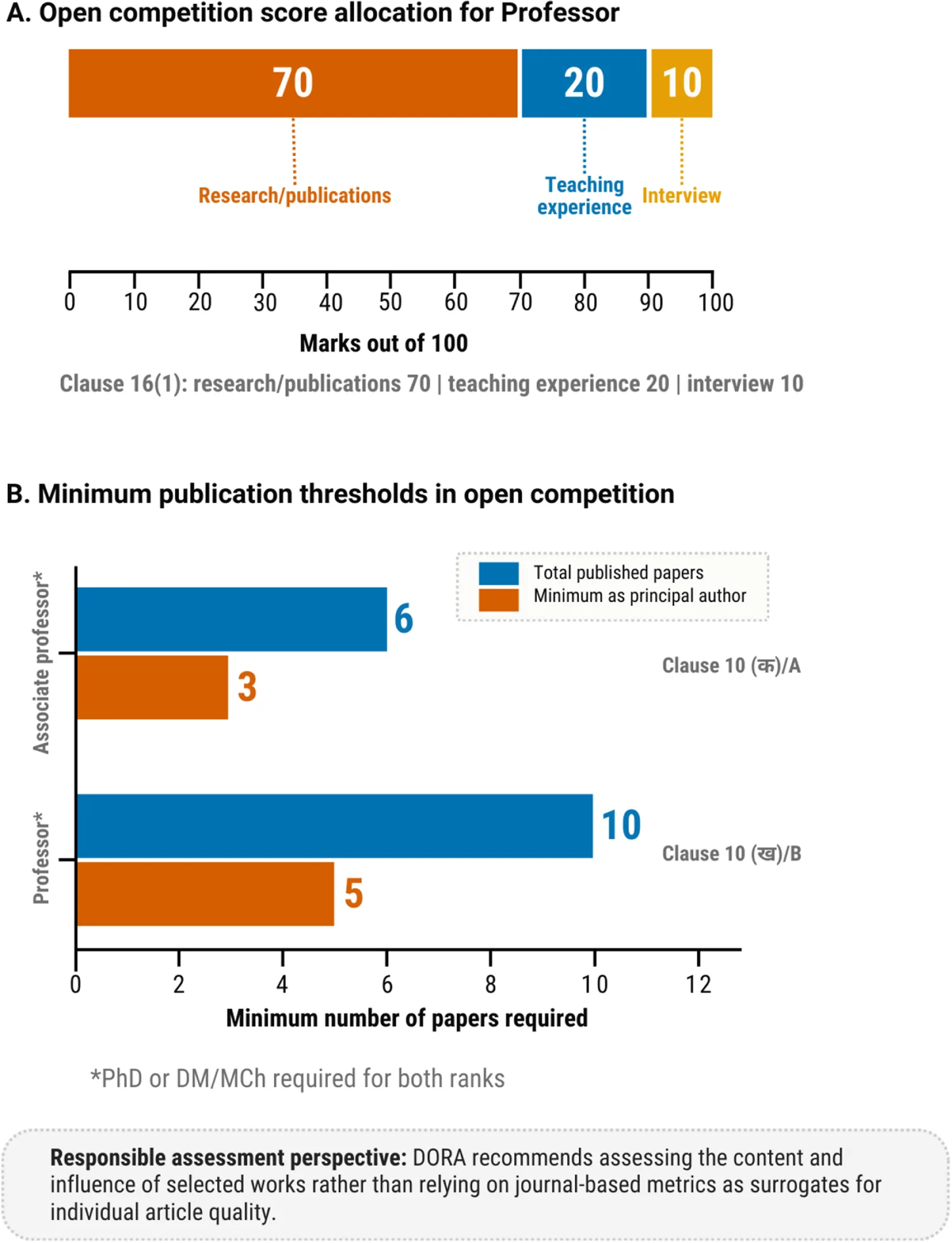
Promotion scoring structure and minimum publication thresholds at Tribhuvan University. (a) Score allocation for open-competition candidates (out of 100 marks), per Clause 16(1) of the Regulations: research and publications, 70 marks; teaching experience, 20; interview, 10. (b) Minimum number of published articles required for open competition at Associate Professor (Clause 10 क/A) and Professor (Clause 10 ख/B) ranks; total articles required and the minimum that must carry Principal Authorship are shown. A PhD or DM/MCh is required for both ranks. DORA, the San Francisco Declaration on Research Assessment.

TU has, to its credit, implemented several commendable integrity safeguards. A strict 20% similarity-index threshold excludes plagiarized work from evaluation. Publications in journals identified as predatory or fraudulent are entirely invalidated, with the TU Service Commission actively maintaining and publishing updated exclusion lists^[^4,5^]^. Manuscripts accepted but not published before the advertisement date are ineligible. The recognition of the DM and MCh degrees as equivalent to the PhD, alongside lowered internal promotion pass thresholds for Institute of Medicine faculty (55% versus the standard 65% for the rank of Professor), acknowledges the heavy clinical burden borne by medical academics[4].

## Why the structure carries risk

Despite these safeguards, the architecture carries significant risks that generalize well beyond any single institution. A 70-point allocation to publications compels candidates to optimize output for volume and Principal Authorship rather than for scientific contribution. Where large-scale clinical trials are logistically and financially prohibitive, this incentive structure may encourage the proliferation of smaller, fragmented, and methodologically weak publications^[^2,6^]^. Fractional authorship scoring penalizes collaborative research, the very model modern medical science demands, by rendering middle-authorship contributions nearly worthless toward meeting promotion thresholds. This can create perverse incentives to pursue isolated, lower-impact studies where first authorship is guaranteed, fostering conditions conducive to honorary and ghost authorship[7].

Reliance on journal-tier classification to assign individual article scores contravenes the San Francisco Declaration on Research Assessment (DORA), which holds that journal-based metrics must not serve as surrogate measures of individual article quality in promotion decisions[8]. By awarding higher marks for Class A (SCImago-indexed) journals than for regional outlets, such a system may discourage locally relevant public health research suited for national journals. The mathematical allocation of authorship points also does not align with the International Committee of Medical Journal Editors’ (ICMJE) four-part criteria for responsible authorship, which prioritize substantive intellectual contribution over positional order[9].

These challenges are not unique to Nepal. Across low- and middle-income countries, similar metric-driven frameworks risk precipitating the “Cobra Effect,” in which incentive structures designed to elevate research quality inadvertently degrade it[6]. When institutional advancement functions as a bounty on publication counts, researchers rationally optimize for the metric rather than the science. This is the central, generalizable lesson that the TU example makes visible.

## Potential solutions

Reforming such systems requires institutional courage. TU’s existing anti-plagiarism and anti-predatory safeguards demonstrate that meaningful regulatory action is achievable. Building on this foundation, institutions operating comparable frameworks should consider a phased transition toward DORA-aligned evaluation: replacing rigid volume thresholds with curated portfolio assessment, in which candidates submit a smaller number of their strongest works for independent evaluation of methodological rigor and societal impact (Fig. 1). Authorship evaluation should incorporate the ICMJE criteria so that credit reflects genuine intellectual contribution. The Committee on Publication Ethics guidelines further urge institutions to ensure that promotion systems do not inadvertently create perverse incentives for redundant publication or authorship manipulation[10].

Research ecosystems across the region are at a pivotal juncture. Reforms of the kind outlined here could serve as a regional exemplar, demonstrating that rigorous research assessment need not rely on counting papers but can instead reward the quality, integrity, and relevance of scholarship. The stakes are not merely institutional; they bear directly on the quality of evidence that informs health policy across South Asia and comparable settings.

## Data Availability

All sources analyzed in this article are publicly available and cited in the reference list. No new datasets were generated or analyzed.
